# Application of environmental DNA metabarcoding and quantitative PCR to detect blooming jellyfish in a temperate bay of northern China

**DOI:** 10.1002/ece3.10669

**Published:** 2023-10-31

**Authors:** Saijun Peng, Lei Wang, Yuanqing Ma, Lijing Ye, Chaowei Hou, Yongliang Liu, Yongxue Li, Tingting Sun, Jianmin Zhao, Zhijun Dong

**Affiliations:** ^1^ Muping Coastal Environment Research Station Yantai Institute of Coastal Zone Research, Chinese Academy of Sciences Yantai Shandong China; ^2^ University of Chinese Academy of Sciences Beijing China; ^3^ Shandong Key Laboratory of Marine Ecological Restoration Shandong Marine Resource and Environment Research Institute Yantai Shandong China

**Keywords:** *Aurelia coerulea*, environmental DNA metabarcoding, jellyfish blooms, quantitative PCR

## Abstract

Frequently occurring jellyfish blooms have severe impacts on the socioeconomics of coastal areas, which stress the importance of early detection and assessments of blooming jellyfish taxa. Environmental DNA (eDNA) techniques (quantitative PCR and eDNA metabarcoding) have the advantage of high sensitivity and are an emerging powerful tool for investigations of target species. However, a comprehensive analysis of the biodiversity and biomass of jellyfish taxa in the target area by combining the two eDNA techniques is still lacking. Here, we developed eDNA metabarcoding and quantitative PCR for the detection and assessment of jellyfish taxa in the temperate Yantai Sishili Bay (YSB) and estimated the spatial distribution of *Aurelia coerulea*. Species‐specific quantitative PCR assays targeting the mitochondrial cytochrome c oxidase subunit I gene of *A. coerulea* were developed. Additionally, eDNA metabarcoding based on the mitochondrial 16S rDNA sequences identified six jellyfish species in YSB. Moreover, our results indicate that *A. coerulea* aggregations were more likely to occur in the inner part of the bay than in the outer part, and they gathered in the bottom layer of seawater rather than in the surface layer. Our results demonstrate the potential of two eDNA techniques in jellyfish biomass investigation and jellyfish taxa detection. These eDNA techniques may contribute to the discovery of jellyfish aggregation so as to achieve early warning of large‐scale jellyfish blooms in coastal areas.

## Graphical abstract

We developed eDNA metabarcoding and quantitative PCR for the detection and assessment of jellyfish taxa in the temperate Yantai Sishili Bay (YSB) and estimated the spatial distribution of *Aurelia coerulea*. Our results demonstrate the potential of two eDNA techniques in jellyfish biomass investigation and jellyfish taxa detecting. These eDNA techniques may contribute to the discovery of jellyfish aggregation so as to achieve early warning of large‐scale jellyfish blooms on coastal areas.

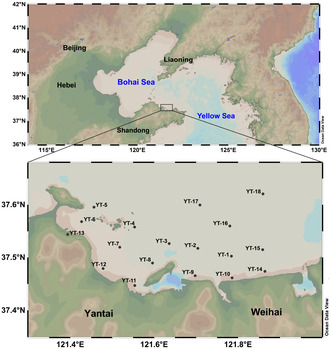

## 1 INTRODUCTION

Jellies are gelatinous zooplankton belonging to the phyla Cnidaria and Ctenophora. The prevalence of jellyfish blooms has received extensive concern, and blooming jellyfish were recorded as causing blockage of nuclear power plants (Wang et al., 2023), threatening the survival of other marine animals (Baxter et al., 2011) and imposing huge economic losses on fisheries and tourism (Baumann & Schernewski, 2012; Conley & Sutherland, 2015). Biological invasions by marine jellyfish are also of increasing concern for biodiversity conservation worldwide (Giallongo et al., 2021; Govindarajan & Carman, 2016; Stampar et al., 2020; van Walraven et al., 2017). To reduce the harmful effects of jellyfish blooms, it is imperative to accurately identify jellyfish species and timely monitoring and early detection of jellyfish population dynamics.

Most jellyfish species have metagenic life cycles, where conspicuous pelagic medusae are the focus of many ecological investigations (Lucas et al., 2014). Tiny and cryptic stages (e.g., planulae, polyps and ephyrae) of jellyfish, critical periods for population expansion, are difficult to detect and identify in situ. Additionally, jellyfish samples have phenotypic plasticity characteristics as well as fragile tissues, and there are cryptic species, which pose challenges to traditional morphological identification. The emergence of the DNA barcoding technique promoted the development of jellyfish species identification and population dynamics investigation. The mitochondrial cytochrome c oxidase subunit I (COI), 16S rDNA, and nuclear ITS genes have been used to perform species identification and phylogenetic analysis of scyphozoans and hydrozoans (Ramšak et al., 2012; Scorrano et al., 2017; Zheng et al., 2014). However, the conventional DNA barcoding technique relies on the collection of target samples and cannot evaluate the community composition in the designated area.

Environmental DNA (eDNA) techniques, which detect the DNA fragments directly extracted from the environment including DNA from living cells shed by organisms and extracellular DNA freed from cells after an organism dies (Nielsen et al., 2007), have emerged as a potential powerful tool to assess aquatic community structures in a specified area. A species‐specific quantitative PCR method utilizes targeted primers focusing on the detection of a few targeted species (e.g., Bolte et al., 2021; Gaynor et al., 2017; Minamoto et al., 2017; Ogata et al., 2021; Sathirapongsasuti et al., 2021; Takahashi et al., 2020; Takasu et al., 2019; Wang et al., 2021). In contrast, the detection of multiple species can be undertaken through general metabarcoding using conserved primers (e.g., Alexander et al., 2020; Ames et al., 2021; Beentjes et al., 2022; Clark et al., 2020; Euclide et al., 2021; Pappalardo et al., 2021), which is advantageous in biodiversity surveys. Recently, several studies combined targeted PCR and general metabarcoding and carried out comprehensive ecological surveys of target species from both qualitative and quantitative levels, as exemplified in research on the bighead carp *Hypophthalmichthys nobilis* (Simmons et al., 2016), the great crested newt *Triturus cristatus* (Harper et al., 2018), the Mediterranean fanworm *Sabella spallanzanii* (Wood et al., 2019), the broadly invasive carpet sea squirt *Didemnum vexillum* (Gargan et al., 2022) and diverse fish species (McCarthy et al., 2022; Pont et al., 2022; Wu et al., 2022; Yu et al., 2022). Most reports supported the higher sensitivity and robustness of targeted PCR over general metabarcoding techniques. Conversely, individual studies showed higher or equivalent sensitivity in general metabarcoding (McCarthy et al., 2022; Westfall et al., 2021). Jellyfish taxa, however, have been investigated only by using a single eDNA approach (Ames et al., 2021; Bolte et al., 2021; Gaynor et al., 2017; Minamoto et al., 2017; Ogata et al., 2021; Takahashi et al., 2020), and the combined application of two eDNA methods is still lacking.

In recent years, blooms of the moon jellyfish *Aurelia coerulea* have occurred frequently in summer in Yantai Sishili Bay (YSB), a typical temperate bay located in the northern Yellow Sea (Dong et al., 2012; Peng et al., 2021). YSB is an important shallow sea aquaculture area and has established various fishery biological aquaculture systems, mainly scallops. The damage of jellyfish blooms in the commercial fishery and aquaculture has been a concern in previous reports (Conley & Sutherland, 2015; Richardson et al., 2009). Therefore, the monitoring and prevention of jellyfish blooms in the YSB area are of great value for the stable development of aquaculture. Herein, eDNA samples of seawater and sediments in YSB were collected in July and August 2022. The concentration of the *A. coerulea* eDNA was specifically quantified by fluorescence quantitative PCR (qPCR) of the mitochondrial COI gene sequence from eDNA in YSB. Simultaneously, the eDNA metabarcoding method based on the mitochondrial 16S rDNA sequence was used to detect and identify jellyfish taxa in YSB. The application potential of eDNA metabarcoding and qPCR in seawater and sediment environments for the detection and assessment of jellyfish in YSB was analyzed. This study is the first to combine eDNA metabarcoding and qPCR methods in jellyfish detection, which supports efficient jellyfish ecological survey.

## 2 MATERIALS AND METHODS

### 2.1 Field sample collection and processing

**FIGURE 1:**
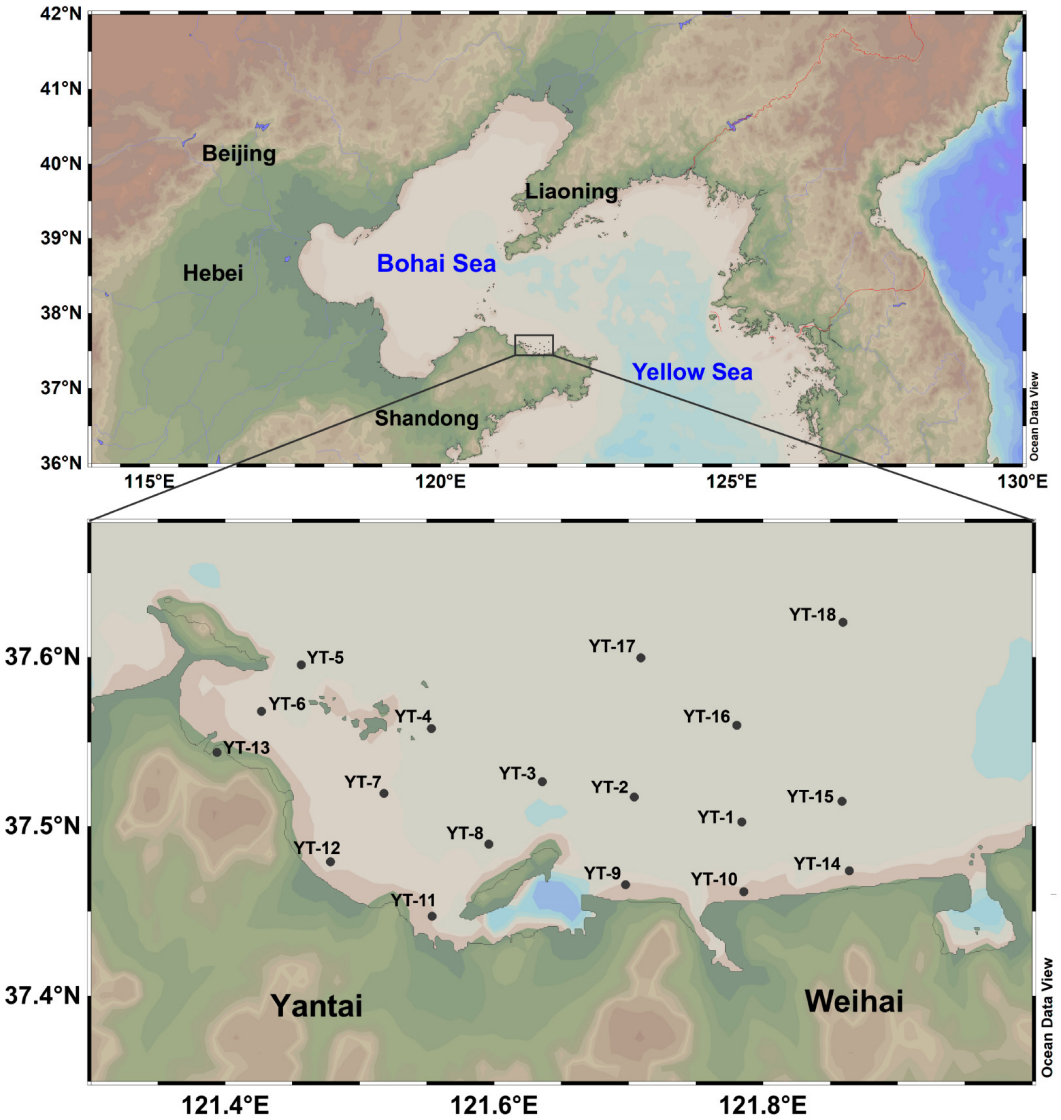
Sampling locations in Yantai Sishili Bay.

Two cruises were conducted in YSB from July 18 to 21 and August 16 to 18, 2022. Eighteen stations (YT‐1–18) were surveyed in July, and 17 stations in August (except station YT‐14) (Figure 1). YT‐1–15 were at the inner parts of the bay, whereas YT‐16–18 were at the outer parts. At each station, 1 L of surface and bottom layer seawater were collected by a water sampler in sterile 1‐L plastic bottles, and the sampling depths were recorded, as shown in Table S1. Each water sample (1 L) was filtered through a 0.7‐μm GF/F filter membrane (Whatman) (Minamoto et al., 2017; Takahashi et al., 2020) and stored in 2‐mL sterile freezing tubes. In total, 70 membrane samples of surface (*n* = 35) and bottom (*n* = 35) layer seawater were obtained during the two cruises. To avoid foreign DNA contamination, one negative control was set for each filtration, that is, 1 L of distilled water was filtered at each station. Sediment samples were collected from each station using a bottom sampler, dug with a sterile disposable syringe, and placed in 50‐mL sterile centrifuge tubes. A total of 35 sediment samples were obtained during the two cruises. All water sample membranes and sediment samples were temporarily placed in liquid nitrogen until they were returned to the laboratory and quickly transferred to a −80°C refrigerator. Filtering devices and samplers were bleached after every sampling with 10% sodium hypochlorite for 5 min and rinsed with Milli‐Q water.

The seawater environmental factors, including seawater temperature, salinity, pH, dissolved oxygen (DO), pressure (Press), total chlorophyll (Chl) concentration and depth, were measured in the field with a YSI EXO2 multiparameter water quality analyzer (YSI, America). Based on Millero et al. (1980), the seawater density of each station (σt, g/cm^3^) was calculated from the seawater temperature, salinity and pressure data (Table S1).

### 2.2 Laboratory degradation experiment

*Aurelia coerulea* medusae were collected in the coastal waters of YSB in September 2022 and kept in a laboratory mariculture tank for 3 days to acclimate. After acclimation, three medusae (individuals without planulae in the gonads), approximately 15 cm in diameter, were placed separately in three 5‐L culture tanks containing 4 L of sterile artificial seawater (pre‐filtered with 0.22 μm MCE filter membranes), as three replicates. The jellyfish were allowed to move freely and were removed after 24 h. Subsequently, seawater samples (1 L each time) of Days 0, 5, and 10 were collected from each tank and filtered on 0.7‐μm GF/F filter membranes (Whatman). A total of nine membrane samples were obtained. Membranes were stored in 2‐mL sterile freezing tubes, treated with liquid nitrogen for 30 min and then quickly transferred to a −80°C refrigerator.

### 2.3 eDNA extraction

The eDNA extraction of GF/F filter membranes from field collection (*n* = 70) and the laboratory experiment (*n* = 9) were performed using DNeasy Blood & Tissue Kits (Qiagen) according to Takahashi et al. (2020), with minor modifications. Each membrane was placed in the suspended part of a Salivette tube (Sarstedt). Then, a 440‐μL solution containing 40 μL of Proteinase K and 400 μL of AL buffer was put on the membrane, and the tube was incubated at 56°C for 1 h. The liquid held in the membrane was collected by centrifuging for 3 min at 5000 × *g*. TE buffer (200 μL) was put on the membrane and centrifuged again for 3 min at 5000 × *g*. Subsequently, 200 μL of AL buffer and 600 μL of ethanol (100%) were added to the collected liquid, and the mixture was transferred to a spin column. Then, we followed the manufacturer's instructions and eluted in an 80‐μL AE buffer before preserving at −20°C. A negative control, that is one new blank filter membrane, was set up during the eDNA extraction process to detect any contamination.

The eDNA extraction of sediments from field collection (*n* = 35) was performed using a DNeasy PowerSoil Pro Kit (Qiagen). Approximately 0.25 g of sediment was weighed per sample for extraction. Then, we followed the manufacturer's instructions and eluted in an 80‐μL Solution C6 before preserving at −20°C. Once again, a negative control, that is 0.25 g of Milli‐Q water, was set up during the eDNA extraction process to detect any contamination.

### 2.4 Quantitative PCR assay

For *A. coerulea* in eDNA samples, the specific primers for qPCR targeting a 172‐bp region of the mitochondrial COI gene based on the mitochondrial genome (NC_046792) downloaded from the NCBI database were designed using Primer 3.0 software as follows: QACOF 5′‐AAGCATTTATGCCCGACGGAA‐3′; QACOR 5′‐TCTGAGCCAACACTTCCTTCAA‐3′. The specificity of the primers was verified by the Primer‐BLAST of the NCBI database according to the default settings. Each qPCR was run on an ABI 7500 Fast platform using SYBR Green fluorescence quantitative PCR and consisted of 10‐μL SYBR Green I mix, 0.5 μL each of forward and reverse primers (10 μM), 1 μL template eDNA and 8 μL of ddH_2_O for a final reaction volume of 20 μL. The qPCR reaction conditions were as follows: hold for 10 min at 95°C, then 45 cycles of 30 s at 95°C, 15 s at 56°C and 35 s at 72°C. At the end of the qPCR run, a melt curve analysis was conducted to confirm there was no contamination (15 s at 95°C, 1 min at 60°C, 30 s at 95°C and 15 s at 60°C). Each sample was run in triplicate, with each plate including three negative controls (i.e., 20 μL of ddH_2_O). Standard curves were constructed using a plasmid containing the *A. coerulea* target gene and a dilution series of 10^−1^–10^−7^ of the original concentration in triplicate. The amplification efficiency of all qPCR reactions was above 80%, and the correlation coefficient (*R* ^2^) was greater than 99%.

### 2.5 eDNA metabarcoding assay

Seven pairs of published primers were used for PCR amplification attempts of eDNA (see Table S2 for details). In this study, the amplification efficiency was evaluated according to the band brightness and PCR product concentration to select the most suitable primer pair for eDNA samples.

PCR amplification was performed using a 20‐μL reaction system of TransStart FastPfu DNA Polymerase (TransGen AP221‐02) including 4 μL 5 × FastPfu Buffer, 2 μL 2.5 mM dNTPs, 0.8 μL each of forward and reverse primers with barcodes (5 μM), 0.4 μL FastPfu Polymerase, 0.2 μL BSA, 2 μL template DNA and 9.8 μL ddH_2_O. The following programs were run on the ABI GeneAmp® 9700 PCR instrument: initial denaturation at 95°C for 3 min and 37 cycles of 95°C for 30 s, 60°C for 30 s and 72°C for 45 s, followed by a final extension executed at 72°C for 10 min. Three replicates were used for each sample. The PCR products from the same sample were mixed and detected by electrophoresis in a 2% (w/v) agarose gel. Subsequently, the PCR products were recovered with an AxyPrep DNA gel recovery kit (AXYGEN), eluted with Tris–HCl buffer and detected again on 2% agarose gel electrophoresis. The PCR amplicons of each sample were quantified by the QuantiFluor™‐ST Blue Fluorescence Quantification System (Promega) and then normalized to equimolar amounts. The amplicon libraries were generated using TruSeq™ DNA Sample Prep Kit (Illumina) and paired‐end sequenced (2 × 300 bp) on a MiSeq platform at Majorbio Bio‐Pharm Technology Co., Ltd.

The paired‐end reads obtained from MiSeq high‐throughput sequencing of 48 eDNA samples were merged into consensus sequences with FLASH (version 1.2.11) (Magoč & Salzberg, 2011) and then treated to remove sequences with a mismatch ratio above 0.2. The merged sequences were quality‐filtered to obtain optimized sequences using QIIME v1.9.1 (Caporaso et al., 2010) with the following criteria: exact barcode matching and two nucleotides mismatch in primer matching. Operational taxonomic units (OTUs) were clustered with a 97% sequence similarity cutoff using UPARSE (Edgar, 2013), and chimeric sequences were identified and removed using UCHIME (Edgar et al., 2011). The taxonomy of each sequence was analyzed by BLAST (*E*‐value = 10^−5^) against the Nucleotide Sequence Database (nt_v20210917) of the NCBI database. Singleton OTUs and OTUs being classified as other domains (except for Eukaryota) or kingdoms (except for Metazoa) were removed because of the nonspecific amplification of primers. All samples were rarefied to the sequence number corresponding to the sample with the least sequences (4693 sequences) before downstream analyses.

### 2.6 Data processing and statistical analysis

Ocean data view software was used to visualize maps of sampling stations and the concentration of *A. coerulea* eDNA measured by qPCR. To compare the concentration of *A. coerulea* eDNA between sediment and seawater samples, the gene copy numbers for seawater samples were converted to the same unit as for sediment samples, that is, copies/g, based on the seawater density obtained in the previous step (Table S1). Origin 95 software was used to show the line chart of the concentration of *A. coerulea* eDNA in the laboratory degradation experiment.

Kruskal–Wallis nonparametric tests in SPSS Statistics software 25 (IBM Corporation) were used to test the differences in seawater environmental factors and the differences in the concentration of *A. coerulea* eDNA between Days 0, 5 and 10 in the laboratory experiment and among various stations in the field. The Mann–Whitney nonparametric test was used to analyze the differences in the concentration of *A. coerulea* eDNA between two depths (surface and bottom layer seawater) and two environments (seawater and sediment). A Spearman rank correlation analysis was used to identify the correlation between the concentration of *A. coerulea* eDNA and five environmental indicators (temperature, DO, salinity, pH and Chl) in surface and bottom seawater samples in July and August, respectively.

## 3 RESULTS

### 3.1 Environmental parameters of seawater

Marine hydrographic information on temperature, DO, pH, salinity and Chl measured in situ during field sampling at 18 stations in July and 17 stations in August 2022 (Figure 1) in YSB are shown in Table 1 and in Figures S1 and S2. Both in July and August, the temperature, DO and pH were significantly higher for surface than bottom seawater (Kruskal–Wallis test, *p* < .01). Higher Chl in the surface layer than in the bottom layer was detected in August (*p* < .01), while Chl in the two seawater layers was relatively consistent in July (*p* > .05). However, salinity exhibited a unique pattern, being higher at the bottom of the bay than at the surface in July (*p* < .01) and having no significant difference in August (*p* = .059). Between the two cruises, temperature and salinity were significantly different (Kruskal–Wallis test, *p* < .01), and no significant difference was identified in pH, Chl and DO (*p* > .05). Specifically, August showed significantly higher temperatures and lower salinity than July. Coastal stations (YT‐9–14) generally had higher water temperatures, DO and Chl.

**TABLE 1:** Environmental characteristics of Yantai Sishili Bay (mean ± SE).

Cruises	Layer	Depth (m)	DO (mg/L)	T (°C)	Chl (μg/L)	Salinity (ppt)	pH
July	Surface	1.07 ± 0.28	9.70 ± 1.60	23.74 ± 0.87	5.59 ± 4.55	29.93 ± 0.43	8.21 ± 0.12
	Bottom	14.37 ± 4.59	5.93 ± 1.60	20.98 ± 0.98	4.93 ± 10.18	30.52 ± 0.16	7.92 ± 0.12
August	Surface	1.59 ± 0.22	7.73 ± 1.67	25.44 ± 0.69	9.39 ± 14.59	28.62 ± 1.20	8.14 ± 0.10
	Bottom	14.91 ± 4.89	4.00 ± 1.35	23.66 ± 0.71	1.93 ± 3.85	29.68 ± 0.45	7.87 ± 0.12

### 3.2 Quantitative PCR for *Aurelia coerulea* detection in the laboratory

**FIGURE 2:**
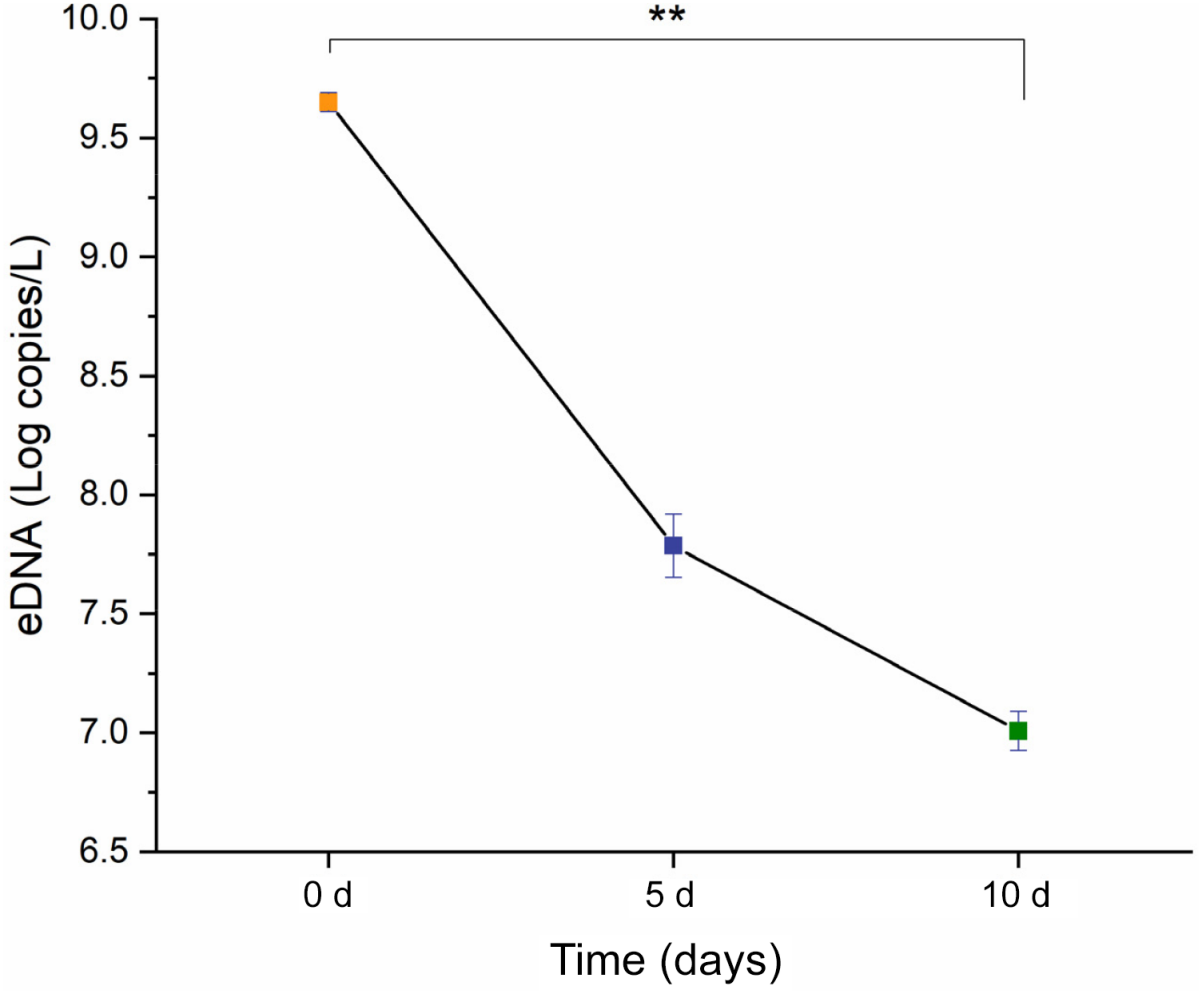
Concentration of *Aurelia coerulea* eDNA in the degradation experiment. “**” indicates highly significant differences (*p* = .007).

*A. coerulea*‐specific primers were developed, and their validity and sensitivity were demonstrated in a laboratory degradation experiment. After the removal of jellyfish, the COI gene of *A. coerulea* in the tanks was still detected after 10 days, with 4.49 × 10^9^ ± 4.27 × 10^8^ copies/L on Day 0, 6.28 × 10^7^ ± 1.76 × 10^7^ copies/L on Day 5 and 1.03 × 10^7^ ± 1.87 × 10^6^ copies/L on Day 10. This shows the stability of jellyfish eDNA in a seawater environment (Figure 2). A Kruskal–Wallis test revealed that the concentration of *A. coerulea* eDNA in seawater significantly decreased between Day 0 and Day 10 (*p* < .01), whereas no significant difference was identified between Day 0 and day 5 or between Day 5 and Day 10 (*p* > .05). The laboratory experiment proved the feasibility of applying qPCR to population identification of the blooming jellyfish *A. coerulea*.

### 3.3 Quantitative PCR for *Aurelia coerulea* detection in the field

In total, 88 of the 105 eDNA samples from YSB were positive for *A. coerulea* based on the qPCR assay. The detection rates (presence/all) of *A. coerulea* in sediment were the highest at 100%, followed by those in bottom seawater at 97.14%. In contrast, *A. coerulea* in surface seawater had lower detection rates of 54.29%. For the two cruises, the detection rates of *A. coerulea* by the qPCR method were comparable in July and August, at 83.33% and 84.31%, respectively.

**FIGURE 3:**
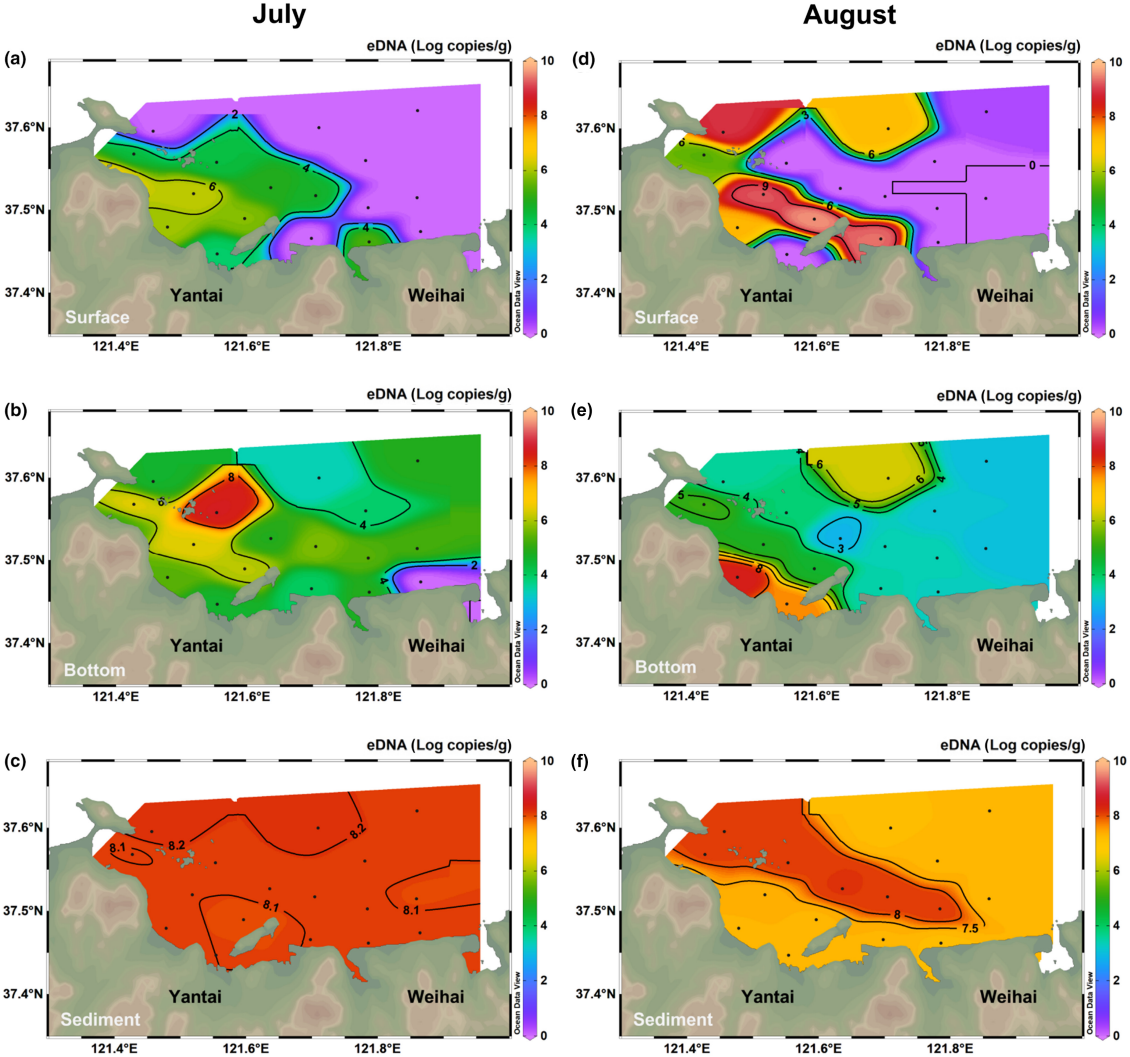
Spatial variations in the concentration of *Aurelia coerulea* eDNA in Yantai Sishili Bay. (a–c) July concentration of *A. coerulea* eDNA in the surface seawater, bottom seawater and sediment, respectively; (d–f) August concentration of *A. coerulea* eDNA in the surface seawater, bottom seawater and sediment, respectively.

In the surface seawater for July, there were significant differences in the concentration of *A. coerulea* eDNA among the stations (Kruskal–Wallis test, *p* < .01), but no significant difference in paired comparisons. The highest concentration of *A. coerulea* eDNA was 6.10 × 10^9^ ± 3.09 × 10^7^ copies/L at YT‐13. Eight stations (YT‐1, 5, 9 and 14–18) were negative for the detection of *A. coerulea* (Figure 3a). In the bottom seawater for July, various stations contained significant differences in the concentration of *A. coerulea* eDNA (Kruskal–Wallis test, *p* < .01), and the differences were mainly reflected between stations YT‐14 and YT‐4 or YT‐6, and between YT‐17 and YT‐4. The highest concentration was 3.95 × 10^11^ ± 1.81 × 10^11^ copies/L in YT‐4, followed by YT‐6 (2.96 × 10^9^ ± 2.05 × 10^8^ copies/L). *A. coerulea* COI gene was undetectable in YT‐14, whereas YT‐17 had the lowest concentration (2.52 × 10^6^ ± 2.39 × 10^5^ copies/L) (Figure 3b). In the sediment sampled in July, the difference was marginal among the stations (Kruskal–Wallis test, *p* > .05), in which the concentration of *A. coerulea* eDNA in YT‐5 (1.73 × 10^8^ ± 1.8 × 10^7^ copies/g) was slightly higher than that of other stations (Figure 3c).

In the surface seawater sampled in August, the concentration of *A. coerulea* eDNA among the stations was significantly different (Kruskal–Wallis test, *p* < .01); however, no significant difference was found in paired comparisons. The highest concentration was 4.48 × 10^12^ ± 1.32 × 10^12^ copies/L in YT‐8, followed by YT‐9 (2.65 × 10^12^ ± 1.02 × 10^12^ copies/L) and YT‐7 (1.70 × 10^12^ ± 3.54 × 10^11^ copies/L), whereas no *A. coerulea* COI gene was detected at eight other stations (YT‐1–4, 10, 11, 15, 16) (Figure 3d). In the bottom seawater in August, the differences in the concentration of *A. coerulea* eDNA among the stations were significant (Kruskal–Wallis test, *p* < .01), especially between YT‐12 and YT‐3 or YT‐15 (*p* < .05). YT‐12 (3.89 × 10^11^ ± 2.15 × 10^11^ copies/L) had the most abundant *A. coerulea* eDNA, followed by YT‐11 (5.37 × 10^10^ ± 1.75 × 10^9^ copies/L). The concentrations of *A. coerulea* eDNA in all 17 stations were positive; however, YT‐3 had the lowest concentration of 8.04 × 10^5^ ± 1.93 × 10^5^ copies/L (Figure 3e). In the sediment sampled in August, there were significant differences in the concentration of *A. coerulea* eDNA among the stations (Kruskal–Wallis test, *p* < .01), but no significant difference in paired comparisons. The concentration of *A. coerulea* eDNA at YT‐1–6 was consistently higher than at other stations, with that at YT‐3 being the highest (1.57 × 10^8^ ± 2.66 × 10^7^ copies/g) (Figure 3f). In summary, the fluctuation of *A. coerulea* eDNA concentration in sediment samples was the smallest among the stations, and *A. coerulea* COI gene was detected in all sediment samples; bottom layer seawater samples showed a greater fluctuation in concentration, whereas the surface layer seawater samples fluctuated the most.

Unit conversion was performed before statistically testing the sediment and seawater samples to make them comparable. Mann–Whitney tests indicated that *A. coerulea* eDNA concentration in sediments (copies/g) both in July and August was significantly higher than in the monthly seawater samples (copies/g) (*p* < .01; Figure 3). In terms of seawater depth, *A. coerulea* eDNA in the bottom seawater in July was significantly more abundant than that in the surface seawater (Mann–Whitney test, *p* < .01; Figure 3a,b). In contrast, the concentration of *A. coerulea* eDNA in surface and bottom seawater was relatively similar in August (Mann–Whitney test, *p* > .05; Figure 3d,e). There was no significant difference in the concentration of *A. coerulea* eDNA in the surface seawater between the two cruises (Mann–Whitney test, *p* > .05; Figure 3a,d). However, the concentration of *A. coerulea* eDNA in the bottom seawater and sediments was statistically different and showed a lower pattern in the August cruise (Mann–Whitney test, *p* < .01; Figure 3b,c,e,f).

A Spearman rank correlation analysis (Table 2) implied that the concentration of *A. coerulea* eDNA in the surface water was significantly correlated with Chl in July (*p* < .01), and the Spearman coefficient was 0.619, with a strong correlation. There was no significant correlation between the concentration of *A. coerulea* eDNA and the five environmental factors in the bottom seawater sampled in July and the surface seawater in August (*p* > .05). In contrast, the concentration of *A. coerulea* eDNA in the bottom seawater in August was closely correlated with all five environmental factors (*p* < .01), in which only salinity was negatively correlated, and the correlation coefficients were greater than 0.6, indicating a strong correlation.

**TABLE 2:** Spearman rank correlation analysis between the concentration of *Aurelia coerulea* eDNA and environmental factors in seawater of Yantai Sishili Bay.

Cruises	Layer	DO	Temperature	Chl	Salinity	pH
July	*Surface*					
	Coefficient	0.147	−0.292	0.617	0.062	−0.046
	Significance	0.561	0.24	0.006**	0.808	0.856
	*Bottom*					
	Coefficient	0.191	0.232	0.393	0.138	0.249
	Significance	0.448	0.354	0.107	0.586	0.319
August	*Surface*					
	Coefficient	−0.194	−0.395	0.199	0.07	−0.262
	Significance	0.455	0.117	0.443	0.79	0.31
	*Bottom*					
	Coefficient	0.63	0.689	0.723	−0.621	0.634
	Significance	0.007**	0.002**	0.001**	0.008**	0.006**

** *p* < .01, with an extremely significant correlation.

### 3.4 eDNA metabarcoding for jellyfish detection

After comparative analysis, the mitochondrial 16S primer pair (16S‐H; 16S‐L) (Ender & Schierwater, 2003) had the best eDNA amplification effect on the field samples of YSB. In this study, 48 of the 105 eDNA samples from YSB amplified sufficient product and met the requirements of high‐throughput sequencing. In total, 806,735 raw 16S rRNA gene reads were generated for 48 samples. The number of optimized sequences obtained after being quality‐filtered was 768,490, and the average length was 273 bp. After subsampling each sample to an equal sequencing depth and clustering, 26 OTUs at 97% identity were obtained, with the number of OTUs ranging from 1 to 7 per sample. eDNA metabarcoding sequencing identified 24 metazoan species from 5 phyla, 8 classes, 21 families and 23 genera, and the identification percentage of the target jellyfish blasted with the NT database (Nucleotide Sequence Database) was above 99.62%, which was reliable (Table 3). In the case of layer, the detection rates (presence/all) of *A. coerulea* in sediment were the highest at 65.71% by eDNA metabarcoding. *A. coerulea* in bottom seawater had the second‐highest detection rates at 45.71%, and the lowest in surface seawater at 25.71%. For the two cruises, the detection rates of *A. coerulea* by the eDNA metabarcoding method were 57.41% and 33.33% in July and August, respectively.

**TABLE 3:** Summary of taxa identified by eDNA metabarcoding based on 16S rDNA sequences.

Phylum	Class	Family	Species	Best match in NCBI	
				Identity (%)	Accession nos.
Cnidaria	Scyphozoa	Ulmaridae	*Aurelia coerulea*	100/99.62	MZ061800.1
Cnidaria	Scyphozoa	Rhizostomatidae	*Nemopilema nomurai*	99.64	KY454767.1
Cnidaria	Scyphozoa	Cyaneidae	*Cyanea nozakii*	100	MW832753.1
Cnidaria	Scyphozoa	Cassiopeidae	*Cassiopea xamachana*	100	ON545804.1
Cnidaria	Hydrozoa	Olindiidae	*Craspedacusta sowerbii*	100	MK600507.1
Cnidaria	Hydrozoa	Halicreatidae	*Varitentacula yantaiensis*	100	HM053551.1
Cnidaria	Anthozoa	Sagartiidae	*Sagartia ornata*	99.63	KR051008.1
Annelida	Polychaeta	Amphinomidae	*Paramphinome jeffreysii*	87.23	GQ478121.1
Annelida	Polychaeta	Sabellidae	*Dialychone perkinsi*	83.64	HM800972.1
Annelida	Polychaeta	Spionidae	*Prionospio sexoculata*	86.52	LC595703.1
Annelida	Polychaeta	Cirratulidae	*Aphelochaeta* sp.	89.86	MK970999.1
Annelida	Polychaeta	Cirratulidae	*Chaetozone* sp.	88.75	KX867185.1
Annelida	Polychaeta	Trichobranchidae	*Terebellides shetlandica*	87.96	MT166845.1
Annelida	Polychaeta	Orbiniidae	*Scoloplos acmeceps*	91.42	AY532344.1
Annelida	Polychaeta	Orbiniidae	*Scoloplos armiger*	97.81	AY532343.1
Annelida	Polychaeta	Paraonidae	*Aricidea suecica*	87.80	MH700664.1
Annelida	Polychaeta	Paraonidae	*Levinsenia demiri*	86.74	MH700695.1
Arthropoda	Insecta	Baetidae	*Centroptiloides bifasciata*	85.22	AJ971746.1
Arthropoda	Malacostraca	Dorippidae	*Paradorippe polita*	80.61	AY452777.1
Chordata	Mammalia	Procaviidae	*Dendrohyrax dorsalis*	89.90	MW592432.1
Porifera	Demospongiae	Axinellidae	*Axinella corrugata*	83.24	AY791693.1
Porifera	Demospongiae	Clionaidae	*Cliona* sp.	98.83	AF362004.1
Porifera	Demospongiae	Callyspongiidae	*Callyspongia fallax*	97.82	EU863810.1
Porifera	Demospongiae	Latrunculiidae	*Latrunculia apicalis*	95.24	KC952724.1

**FIGURE 4:**
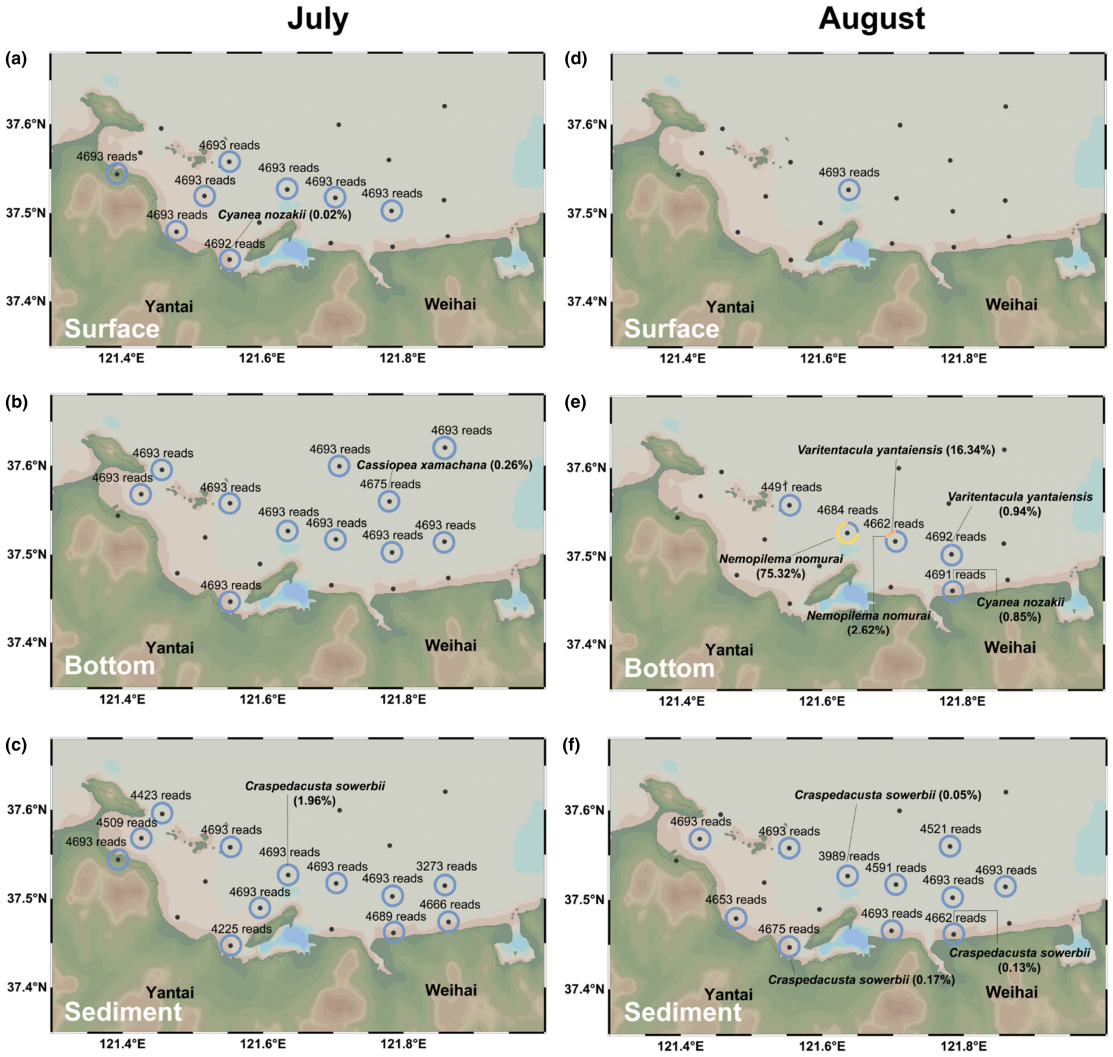
Map depicting the distribution of jellyfish species identified by eDNA metabarcoding based on 16S rDNA sequences in YSB. (a–c) July distribution of jellyfish species in the surface seawater, bottom seawater and sediment, respectively; (d–f) August distribution of jellyfish species in the surface seawater, bottom seawater and sediment, respectively. Circle charts of 48 sequenced samples were plotted to reflect the composition of the medusozoan community at corresponding stations. Unannotated blue rings indicate that *Aurelia coerulea* was the only jellyfish species at that station. If other jellyfish species were identified at a certain station, a specific explanation is given next to the circle, with the relative abundance of the species in parentheses. The number of reads was the total number of medusozoan sequences per station.

The 16S rDNA sequence‐filtered dataset yielded six unique medusozoan taxa, comprising four scyphozoans (*A. coerulea*, *Nemopilema nomurai*, *Cyanea nozakii* and *Cassiopea xamachana*) and two hydrozoans (*Craspedacusta sowerbii* and *Varitentacula yantaiensis*) (Table 3). Considering that the main survey targets of this study were jellyfish, only the community composed of six medusozoan species was analyzed below (Figure 4). The dominant species in 47 samples was *A. coerulea* (relative abundance >81.04%), which was the only identified medusozoan in 37 samples (relative abundance = 100%). Specifically, the abundance of *N. nomurai* was the highest (75.32%) in the bottom seawater samples of YT‐3 in August. *N. nomurai* was also found in low abundance (2.62%) in the bottom seawater samples of the adjacent YT‐2 station in August. *C. nozakii* was only identified in two samples (the surface samples of YT‐11 in July and the bottom samples of YT‐10 in August), and the relative abundances were only 0.02% and 0.85%, respectively. The rare *C. xamachana* was only accidentally identified in the bottom seawater at YT‐16 in July, with a relative abundance of 0.26%. The genetic information of the hydrozoan *C. sowerbii* was sequenced for four sediment samples, including YT‐3 in July (1.96%) and August (0.05%), YT‐10 in August (0.13%), and YT‐11 in August (0.17%). *V. yantaiensis* was detected in bottom seawater samples from two nearby stations in August, YT‐1 (0.94%) and YT‐2 (16.34%).

## 4 DISCUSSION

In recent years, frequent jellyfish blooms have been reported to cause great harm to aquaculture, coastal tourism, and ecological balance (Dong et al., 2012; Li & Liu, 2022; Suzuki et al., 2016, 2019; Wu et al., 2017). As a new marine ecological survey tool, eDNA techniques have the advantages of less harm and high sensitivity, which are expected to provide impetus for the ecological survey of blooming jellyfish. Several studies have confirmed the feasibility and effectiveness of eDNA‐based methods in jellyfish surveys, such as those by Ames et al. (2021), Bolte et al. (2021), Minamoto et al. (2017) and Ogata et al. (2021). Broadly, two main eDNA‐based strategies (qPCR and high‐throughput sequencing) have been employed; however, a co‐application of the two strategies for jellyfish biomonitoring is still lacking. In this study, we discussed the application potential of two eDNA‐based methods (qPCR and eDNA metabarcoding) in detecting and assessing the common blooming jellyfish *A. coerulea* in the seawater and sediment environments of a temperate bay. Additionally, the spatial distribution characteristics of *A. coerulea* in YSB were also estimated based on eDNA metabarcoding and qPCR assays.

The traditional field survey method can lead to underestimation and/or misestimation of target biomass because of sampling omission and empirical species identification (Blackman et al., 2020; Govindarajan et al., 2021). The common advantages of eDNA‐based techniques are manpower conservation, noninvasiveness, environmental friendliness and accurate species identification, which are conducive to large and extensive investigations (Evans et al., 2017; Thomsen et al., 2016; Yamamoto et al., 2017). However, the two primary methods (target qPCR and general metabarcoding) have their own weaknesses. In this study, the species‐specific qPCR showed a higher detection rate and sensitivity than general eDNA metabarcoding assays on *A. coerulea*, consistent with Bylemans et al. (2019), Harper et al. (2018), Schenekar et al. (2020), and Wood et al. (2019). However, considering that the two eDNA techniques used in our study selected two different gene fragments, the comparative analysis was not explored in depth. In the present study, we propose that the species‐specific qPCR method is recommendable when the focus is on a single or a few jellyfish species; however, it largely depends on the specificity of primers and the suitability of the reaction procedure. Limited by the fact that no other medusae except for *A. coerulea* were collected during the cruises, the designed qPCR primers lack the detection of other medusozoans to verify their specific binding and amplification. An eDNA metabarcoding assay may display low detection performance due to false negatives from library preparation failures (Miya et al., 2020; Yu et al., 2022). Nonetheless, an eDNA metabarcoding assay has notable advantages in providing broad‐scale distribution data for multiple species simultaneously in a single analysis (Ames et al., 2021; Euclide et al., 2021; Govindarajan et al., 2021). In this study, eDNA metabarcoding detected six jellyfish taxa, including three common scyphozoans responsible for jellyfish blooms in Chinese seas: *A. coerulea*, *Nemopilema nomurai* and *Cyanea nozakii* (reviewed by Dong et al., 2010). The jellyfish communities in YSB were dominated by *A. coerulea* during the survey period. Unexpectedly, eDNA metabarcoding based on the 16S rDNA detected two jellyfish species with low abundance that are typically not considered to inhabit YSB, *C. xamachana* (a tropical or subtropical species) and *C. sowerbii* (a freshwater species). We suspect that the identification of genetic information does not mean that they inhabit YSB but is primarily due to the introduction of matter from aquariums or rivers. First, the planulae and ephyrae of ornamental jellyfish cultured in coastal aquariums tend to drain into public waters with water changes, causing genetic contamination and even biological invasion (Abe et al., 2017; Enrique‐Navarro & Prieto, 2020). Second, there are many river estuaries in YSB, including the Guangdang River, Xin'an River and Yuniao River, which provide conditions for the inflow of freshwater jellyfish genes (Knudsen et al., 2022; Thomsen et al., 2012). This implies a possible overestimation for the community when using eDNA metabarcoding resulting from genetic contamination. The results indicate the importance of simultaneous traditional visual investigation or trawl sampling as a supplement to avoid possible overestimation, and eDNA methods cannot completely replace traditional surveys in some situations. More specific primers and multiple markers from various regions (e.g., COI, 16S, 12S and 18S rDNA) and of different lengths may be conducive to minimizing assessment bias and enhancing the accuracy of eDNA persistence and state (Alexander et al., 2020; Beentjes et al., 2022; Clark et al., 2020; McCarthy et al., 2022). Ultimately, we conclude that the combination of the two methods should be advocated when funding permits. The combined method will foster a comprehensive understanding of the quantitative distribution of target jellyfish taxa.

Our study focused on estimating the spatial variations of dominant jellyfish *A. coerulea* in YSB based on both qPCR analysis and eDNA metabarcoding sequencing assays. Horizontally, *A. coerulea* were more abundant in the inner part of the bay than in the outer part, which is consistent with a previous trawl survey of *A. coerulea* in YSB (Dong et al., 2012). YSB is a semi‐enclosed bay with relatively slow flow velocity because of the surrounding islands (Kongtong Island and Yangma Island) and the dense aquaculture facilities, which are potential barriers inhibiting water exchange in the area (O'Donncha et al., 2017; Zhou et al., 2021). As a typical zooplankton, *A. coerulea* has poor active swimming ability and mainly relies on the thrust of water flow (Aoki et al., 2012). Thus, the distribution of the assemblages of *A. coerulea* detected in our study was patchy and restricted, most likely because it was a passive response to buoyancy or ocean current rather than an active preferred selection (Suzuki et al., 2018). Moreover, the impact of artificial installations and buildings on jellyfish should also be considered. The coastal and near‐island areas have developed ports or aquaculture. A series of derived marine engineering constructions (such as aquaculture rafts and artificial shorelines) may provide suitable substrates for larval settlement and asexual reproduction (Dong et al., 2018; Holst & Jarms, 2007; Lo et al., 2008; Thé et al., 2020), which is favorable for promoting the emergence of jellyfish blooms.

Furthermore, we found that the concentrations of *A. coerulea* eDNA in the bottom samples were higher in July than in August but similar in surface samples. This means that although the abundance of *A. coerulea* in the upper waters was visually comparable, deeper waters had more jellyfish assemblages in July than in August. A previous study showed that *A. aurita* aggregations were closer to the bottom of Mikawa Bay from April to early July but slowly moved toward the upper layer in subsequent months (Suzuki et al., 2016). Similar dense aggregation on the bottom layer was also discovered in the sea nettle *Chrysaora pacifica* (Minamoto et al., 2017). Moreover, it is noteworthy that there were extremely significant differences in surface and bottom temperatures between the two cruises, implying the possibility of a thermocline. The thermocline has been known to block the vertical passage of particles (Gray & Kingsford, 2003) and may restrict the vertical dispersion of eDNA. Malej et al. (2007) identified that *Aurelia* species are mainly distributed below the thermocline in the marine lakes of Mljet Island. These results revealed one of the advantages of eDNA technology as a tool for species ecological surveys: it is easier to detect hidden distributions which are difficult to be counted by visual investigations.

The concentration of *A. coerulea* eDNA was consistently higher in sediments than in seawater. Our laboratory degradation experiments found significant degradation of eDNA within 10 days after jellyfish removal, similar to the findings of Bolte et al. (2021), Minamoto et al. (2017) and Ogata et al. (2021). In contrast, eDNA in sediments proved to be sustained for a longer period, as even jellyfish blooms from 6 years prior were detected in sediments during a recent study (Ogata et al., 2021). Partial eDNA in sediment samples may be derived from long‐term accumulation or preservation rather than inhabiting organisms. Notably, Ogata et al. (2021) identified jellyfish blooms by eDNA in sediment cores to investigate historical events of jellyfish bloom. However, due to the lack of simultaneous concern for seawater samples, the degree of difference in the concentration of jellyfish eDNA in seawater and sediment environments is unknown, and our research filled this gap. Ultimately, we conclude that sediments are more suitable for counting total target bioaccumulation or investigating resident organisms in the area, whereas seawater reflects target organisms which inhabit the area and recent events, such as blooming and spawning.

## AUTHOR CONTRIBUTIONS

**Saijun Peng:** Data curation (lead); formal analysis (lead); visualization (lead); writing – original draft (lead); writing – review and editing (supporting). **Lei Wang:** Investigation (lead); resources (lead). **Yuanqing Ma:** Conceptualization (supporting); writing – review and editing (supporting). **Lijing Ye:** Resources (supporting). **Chaowei Hou:** Investigation (supporting); resources (supporting). **Yongliang Liu:** Investigation (supporting); resources (supporting). **Yongxue Li:** Writing – review and editing (supporting). **Tingting Sun:** Writing – review and editing (supporting). **Jianmin Zhao:** Conceptualization (supporting); funding acquisition (supporting). **Zhijun Dong:** Conceptualization (lead); data curation (supporting); funding acquisition (lead); validation (lead); writing – review and editing (lead).

## CONFLICT OF INTEREST STATEMENT

The authors declare that they have no conflict of interest.

## Supporting information


Data S1:

## Data Availability

The OTU sequence data generated herein have been uploaded to Science Data Bank under data. doi: 10.57760/sciencedb.07058.
