# Supplementary material for: Application of environmental DNA metabarcoding and quantitative PCR to detect blooming jellyfish in a temperate bay of northern China
Source: Ecol Evol. 2023 Oct 31;13(11):e10669. doi: 10.1002/ece3.10669 (PMC10616739; doi:10.1002/ece3.10669)
Supplement: Supplementary file 1 — Data S1: [file ECE3-13-e10669-s001.docx]

**Appendix:**

Supplementary Table A1: Supplementary environmental indicators and seawater density of the stations in Yantai Sishili Bay.

| **Sites** | **Surface in July** | | | **Bottom in July** | | |
| --- | --- | --- | --- | --- | --- | --- |
|  | **Depth**  **(m)** | **Press**  **(psi)** | **Density**  **(g/cm^3^)** | **Depth**  **(m)** | **Press**  **(psi)** | **Density**  **(g/cm^3^)** |
| YT-1 | 0.88 | 1.27 | 1.0134 | 17.60 | 25.57 | 1.0101 |
| YT-2 | 1.04 | 1.50 | 1.0135 | 17.00 | 24.70 | 1.0104 |
| YT-3 | 1.61 | 2.34 | 1.0133 | 16.00 | 23.24 | 1.0111 |
| YT-4 | 1.53 | 2.21 | 1.0123 | 17.51 | 25.42 | 1.0113 |
| YT-5 | 1.02 | 1.48 | 1.0142 | 16.51 | 23.97 | 1.0116 |
| YT-6 | 1.05 | 1.52 | 1.0121 | 10.36 | 15.03 | 1.0119 |
| YT-7 | 0.94 | 1.36 | 1.0124 | 10.90 | 15.83 | 1.0116 |
| YT-8 | 0.92 | 1.34 | 1.0137 | 13.49 | 19.60 | 1.0107 |
| YT-9 | 1.55 | 2.24 | 1.0139 | 15.27 | 22.17 | 1.0106 |
| YT-10 | 0.92 | 1.33 | 1.0146 | 11.00 | 15.98 | 1.0109 |
| YT-11 | 0.78 | 1.13 | 1.0134 | 5.26 | 7.64 | 1.0129 |
| YT-12 | 1.03 | 1.49 | 1.0119 | 9.10 | 13.21 | 1.0117 |
| YT-13 | 1.51 | 2.20 | 1.0138 | 5.51 | 8.00 | 1.0121 |
| YT-14 | 0.99 | 1.43 | 1.0143 | 19.11 | 27.76 | 1.0101 |
| YT-15 | 0.68 | 0.99 | 1.0134 | 19.52 | 28.36 | 1.0102 |
| YT-16 | 0.80 | 1.15 | 1.0134 | 18.00 | 26.15 | 1.0107 |
| YT-17 | 0.96 | 1.39 | 1.0132 | 17.50 | 25.41 | 1.0109 |
| YT-18 | 1.13 | 1.64 | 1.0132 | 19.01 | 27.61 | 1.0107 |
| **Sites** | **Surface in August** | | | **Bottom in August** | | |
|  | **Depth**  **(m)** | **Press**  **(psi)** | **Density**  **(g/cm^3^)** | **Depth (m)** | **Press**  **(psi)** | **Density**  **(g/cm^3^)** |
| YT-1 | 1.65 | 2.39 | 1.0152 | 18.99 | 27.55 | 1.0128 |
| YT-2 | 1.31 | 1.90 | 1.0154 | 17.50 | 25.39 | 1.0129 |
| YT-3 | 1.30 | 1.88 | 1.0148 | 17.17 | 24.90 | 1.0131 |
| YT-4 | 1.44 | 2.09 | 1.0144 | 18.71 | 27.14 | 1.0136 |
| YT-5 | 1.49 | 2.16 | 1.0144 | 15.55 | 22.55 | 1.0137 |
| YT-6 | 1.26 | 1.82 | 1.0140 | 10.57 | 15.32 | 1.0139 |
| YT-7 | 1.68 | 2.43 | 1.0144 | 11.61 | 16.84 | 1.0137 |
| YT-8 | 1.76 | 2.56 | 1.0149 | 14.02 | 20.34 | 1.0133 |
| YT-9 | 1.26 | 1.82 | 1.0161 | 15.65 | 22.71 | 1.0131 |
| YT-10 | 2.00 | 2.89 | 1.0154 | 15.65 | 22.70 | 1.0130 |
| YT-11 | 1.66 | 2.39 | 1.0171 | 5.02 | 7.26 | 1.0151 |
| YT-12 | 1.60 | 2.31 | 1.0141 | 9.70 | 14.06 | 1.0139 |
| YT-13 | 1.86 | 2.70 | 1.0148 | 5.14 | 7.44 | 1.0142 |
| YT-15 | 1.64 | 2.37 | 1.0155 | 19.95 | 28.95 | 1.0129 |
| YT-16 | 1.63 | 2.36 | 1.0155 | 19.17 | 27.81 | 1.0128 |
| YT-17 | 1.78 | 2.57 | 1.0147 | 18.51 | 26.85 | 1.0128 |
| YT-18 | 1.78 | 2.58 | 1.0149 | 20.51 | 29.76 | 1.0124 |

Supplementary Table A2: Primer information for PCR amplification preliminary experiments of eDNA

| Primer name | Sequences (5’-3’) | Main range | References |
| --- | --- | --- | --- |
| Uni18SF-V4 | AGGGCAAKYCTGGTGCCAG | Eukaryota | Clark et al., 2020 |
| Uni18SR-V4 | CGRCGGTATCTRATCGYCTT |  |  |
| 528F | GCCTCCCTCGCGCCATCAGGCGGTAATTCCAGCTCCAA | Eukaryota | Universal primer |
| 706R | GCCTTGCCAGCCCGATCAGAATCCRAGAATTTCACCTCT |  |  |
| HCO2198 | TAAACTTCAGGGTGACCAAAAAATCA | Metazoan invertebrates | Folmer et al., 1994 |
| LCO1490 | GGTCAACAAATCATAAAGATATTGG |  |  |
| mlCOIintF | GGWACWGGWTGA ACWGTWTAYCCYCC | Metazoa | Leray et al., 2013 |
| jgHCO2198 | TAIACYTCIGGRTGICCRAARAAYCA |  |  |
| med-rnl-F-16S | GACTGTTTACCAAAGACATAGC | Medusozoa | Lawley et al., 2016 |
| med-rnl-R-16S | AAGATAGAAACCTTCCTGTC |  |  |
| med-cox1-F | ACNAAYCAYAAAGATATHGG | Medusozoa | Lawley et al., 2016 |
| med-cox1-R | TGGTGNGCYCANACNATRAANCC |  |  |
| 16S-H | CATAATTCAACATCGAGG | Cnidaria | Ender and Schierwater, 2003 |
| 16S-L | GACTGTTTACCAAAAACATA |  |  |

Supplementary Figure A1: Marine hydrographic maps of Yantai Sishili Bay in July 2022. a-e were the temperature, dissolved oxygen, salinity, pH and chlorophyll concentrations of surface seawater in July; f-j were the temperature, dissolved oxygen, salinity, pH and chlorophyll concentrations of bottom seawater in July.


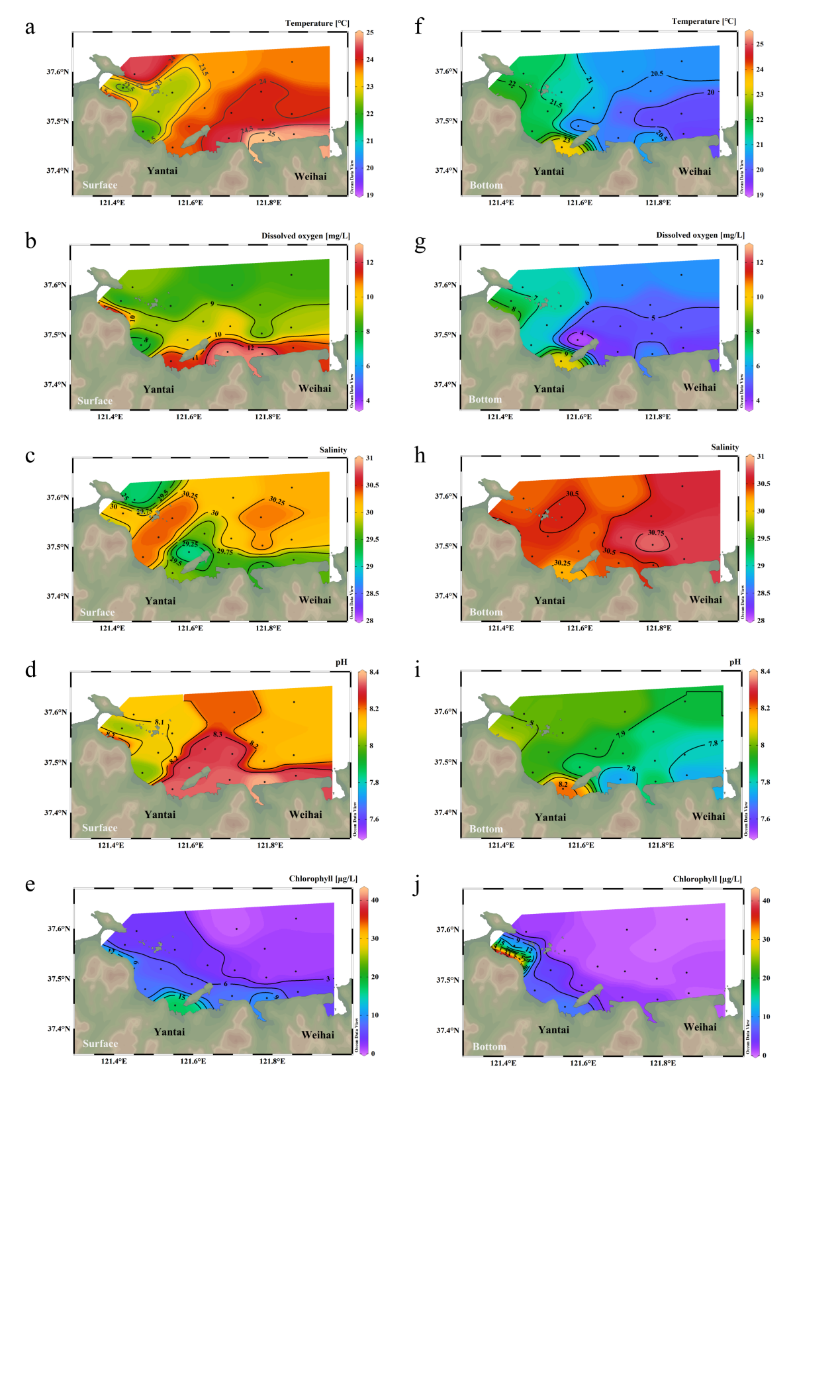


Supplementary Figure A2: Marine hydrographic maps of Yantai Sishili Bay in August 2022. a-e were the temperature, dissolved oxygen, salinity, pH and chlorophyll concentrations of surface seawater in August; f-j were the temperature, dissolved oxygen, salinity, pH and chlorophyll concentrations of bottom seawater in August.


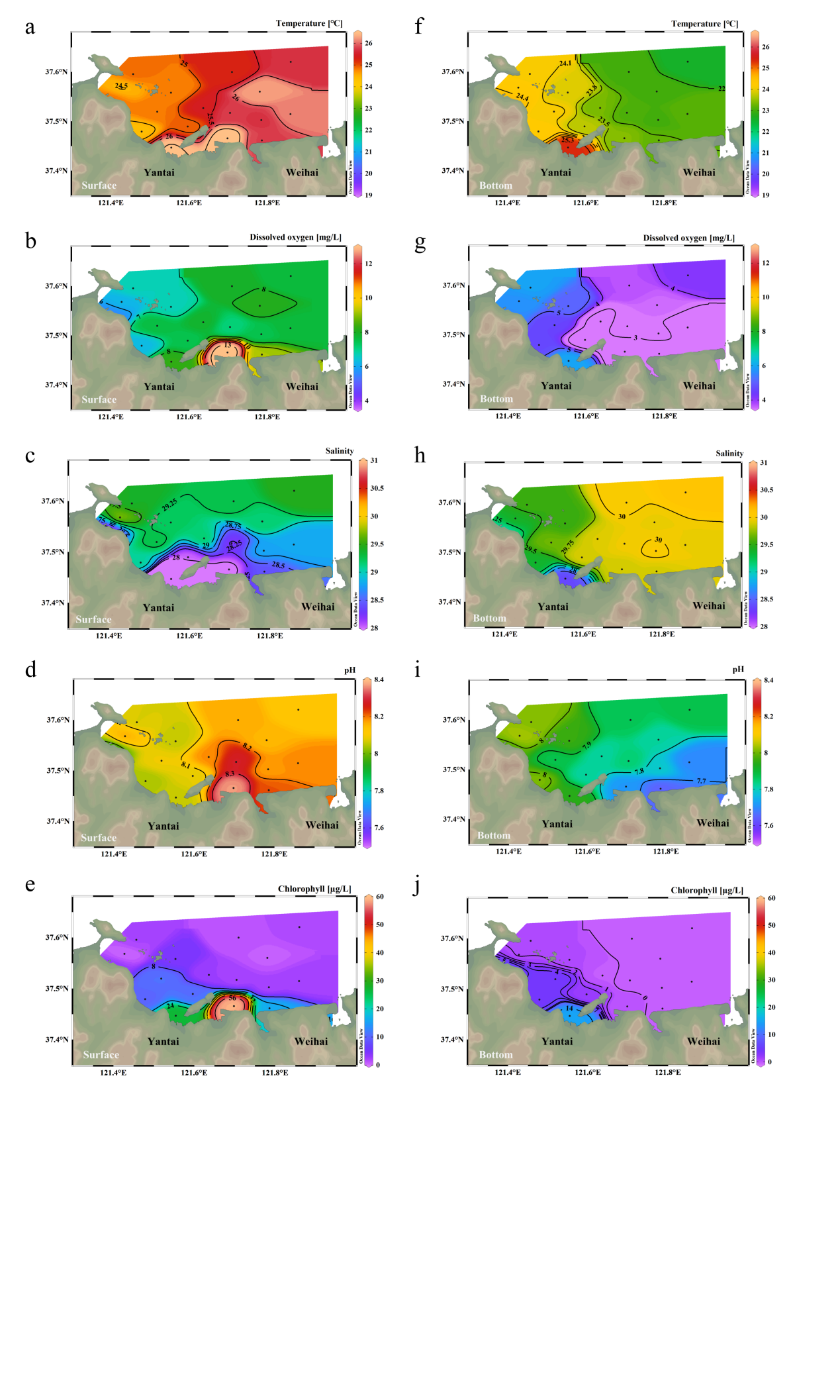


Supplementary References:

Clark, D. E., Pilditch, C. A., Pearman, J. K., Ellis, J. I., & Zaiko, A. (2020). Environmental DNA metabarcoding reveals estuarine benthic community response to nutrient enrichment - Evidence from an *in-situ* experiment.

*Environmental Pollution*, 267, 115472.

Ender, A., & Schierwater, B. (2003). Placozoa are not derived cnidarians: Evidence from molecular morphology. *Molecular Biology and Evolution*, 20(1), 130-134.

Folmer, O., Black, M., Hoeh, W., Lutz, R., & Vrijenhoek, R. (1994). DNA primers for amplification of mitochondrial cytochrome c oxidase subunit I from diverse metazoan invertebrates. *Molecular Marine Biology and Biotechnology*, 3(5), 294-299.

Lawley, J. W., Ames, C. L., Bentlage, B., Yanagihara, A., Goodwill, R., Kayal, E., Hurwitz, K., & Collins, A. G. (2016). Box jellyfish *Alatina alata* has a circumtropical distribution. *Biological Bulletin*, 231(2), 152-169.

Leray, M., Yang, J. Y., Meyer, C. P., Mills, S. C., Agudelo, N., Ranwez, V., Boehm, J. T., & Machida, R. J. (2013). A new versatile primer set targeting a short fragment of the mitochondrial COI region for metabarcoding metazoan diversity: application for characterizing coral reef fish gut contents. *Frontiers in Zoology*, 10, 34.
